# *Bletilla striata* polysaccharide-coated andrographolide nanomicelles for targeted drug delivery to enhance anti-colon cancer efficacy

**DOI:** 10.3389/fimmu.2024.1380229

**Published:** 2024-06-07

**Authors:** Zhongqun Yue, Yue Zhu, Teng Chen, Tingting Feng, Ying Zhou, Jiaojiao Zhang, Ning Zhang, Jing Yang, Gang Luo, Zuhua Wang

**Affiliations:** ^1^ College of Pharmaceutical Sciences, Guizhou University of Traditional Chinese Medicine, Guiyang, China; ^2^ Nano-drug Technology Research Center of Guizhou University of Traditional Chinese Medicine, Guiyang, China; ^3^ College of Food and Health, Zhejiang A&F University, Hangzhou, China; ^4^ School of Acupuncture-Moxibustion and Tuina of Guizhou University of Traditional Chinese Medicine, Guiyang, China; ^5^ Key Laboratory of Medical Microbiology and Parasitology and Key Laboratory of Environmental Pollution Monitoring and Disease Control, Ministry of Education, School of Basic Medical Sciences, Guizhou Medical University, Guiyang, China

**Keywords:** colon cancer, *Bletilla striata* polysaccharide, andrographolide, polymer, nano-micelle

## Abstract

**Background:**

Vitamin E, which is also known as tocopherol, is a compound with a polyphenol structure. Its esterified derivative, Vitamin E succinate (VES), exhibits unique anticancer and healthcare functions as well as immunomodulatory effects. Natural polysaccharides are proved to be a promising material for nano-drug delivery systems, which show excellent biodegradability and biocompatibility. In this study, we employed a novel *bletilla striata* polysaccharide-vitamin E succinate polymer (BSP-VES) micelles to enhance the tumor targeting and anti-colon cancer effect of andrographolide (AG).

**Methods:**

BSP-VES polymer was synthesized through esterification and its structure was confirmed using 1H NMR. AG@BSP-VES was prepared via the dialysis method and the drug loading, entrapment efficiency, stability, and safety were assessed. Furthermore, the tumor targeting ability of AG@BSP-VES was evaluated through targeted cell uptake and *in vivo* imaging. The antitumor activity of AG@BSP-VES was measured *in vitro* using MTT assay, Live&Dead cell staining, and cell scratch test.

**Results:**

In this study, we successfully loaded AG into BSP-VES micelles (AG@BSP-VES), which exhibited good stability, biosafety and sustained release effect. In addition, AG@BSP-VES also showed excellent internalization capability into CT26 cells compared with NCM460 cells *in vitro*. Meanwhile, the specific delivery of AG@BSP-VES micelles into subcutaneous and *in-situ* colon tumors was observed compared with normal colon tissues *in vivo* during the whole experiment process (1–24 h). What’s more, AG@BSP-VES micelles exhibited significant antitumor activities than BSP-VES micelles and free AG.

**Conclusion:**

The study provides a meaningful new idea and method for application in drug delivery system and targeted treatment of colon cancer based on natural polysaccharides.

## 1 Introduction

Colorectal cancer is a prevalent malignancy of the digestive tract and the third most common cancer globally, ranking second in cancer-related deaths (1). Current treatments include surgery and chemotherapy, which are not always effective and can bring about severe side effects, such as poor selectivity and low bio-availability. In addition, while these treatments target cancer cells, they also damage normal human cells, leading to inevitable harm (2). Therefore, the development of tumor-specific drug delivery systems is of clinical significance, and the use of nanodrug delivery systems has solved some of the problems in tumor drug therapy including colon cancer (3). Currently, nanodrug delivery systems commonly include liposomes, polymeric micelles, and nanospheres (4). Among these delivery systems, polymeric micelle is considered a promising strategy for antitumor drug delivery due to its amphiphilic properties and thermodynamic stability (5–7). However, setbacks like low biocompatibility and *in vivo* difficult degradation limit synthetic polymeric micellar transporters its application of micelles (8), and lead researchers to consider natural hydrophilic polymers, such as polysaccharides (9). It is suitable for the development of amphiphilic derivatives due to their low cost, good safety, and easy modification. Polysaccharides covalently combining with hydrophobic groups to form amphiphilic polysaccharide derivatives, can self-assemble nano-sized micelles in water, which can wrap hydrophobic drugs to improve their solubility and bioavailability (10–12). As nanocarriers, phyto-based polysaccharides have unique tumor immunomodulatory activities and excellent biocompatibility to enhance total therapeutic effects.

*Bletilla striata*, a dried orchid bulb, extracted from ‘Shennong’s Herbal Classic ’ (13), in which records its long historical medicinal use. *B. striata* has high medicinal value, including antibacterial, anti-tumor, hemostasis, promoting wound healing, immune regulation, promoting bone marrow hematopoietic and so on. *B. striata* polysaccharide (BSP), the primary active ingredient of *B. striata*, is mainly composed of mannose and glucose, with a mannose to glucose ratio of 2.4:1 or 3:1 (14). In this study, the selected BSP was polymerized through β-glycosidic bond polymerization of mannose and glucose in a ratio of 2.4:1. BSP has excellent antitumor activity that can inhibit proliferation of human cancer lines such as gastric cancer cells (MKN45), ovarian cancer cells (A2780), liver cancer cells (HepG2), and colon cancer (CT26), etc (15–19). In addition, BSP as a novel natural polymer material (20, 21), possesses several advantages, including functional slow release, abundant resources, and reduced drug toxicity. When used as a carrier, it can act as a ‘combination of medicine and adjuvant’ (22, 23). Previous research has demonstrated that BSP can undergo self-assembly to create nano-micelles in aqueous solutions through hydrophobic modification (24, 25). Vitamin E succinate (VES) is a lipid derivative of vitamin E that possesses both the physiological functions of natural vitamin E and unique anti-cancer and immunomodulatory properties. It has emerged as the most widely used raw material for tumor drugs worldwide. Due to its long fat chain and strong hydrophobicity, it can be effectively employed for hydrophobic modification of polysaccharides (26). However, there are currently no research on the formation of polymer micelles through the hydrophobic modification of BSP with VES yet.

Andrographolide (AG) is a diterpene lactone derived from Andrographis paniculata, which has the ability to inhibit cancer cell proliferation and induce cell apoptosis. It mainly inhibits cancer development through intracellular signal transduction mediated by Wnt/β-catenin, mTOR, VEGF, and TRAIL-mediated cell apoptosis (27). Therefore, AG has the potential to become an anti-cancer candidate drug in the treatment of colorectal cancer. However, the clinical application of AG has been limited because of its defects such as low bioavailability and high hydrophobicity. In order to enhance the therapeutic effects of AG, design AG-loaded nanoscale formulations is very important (28).

**Scheme 1:**
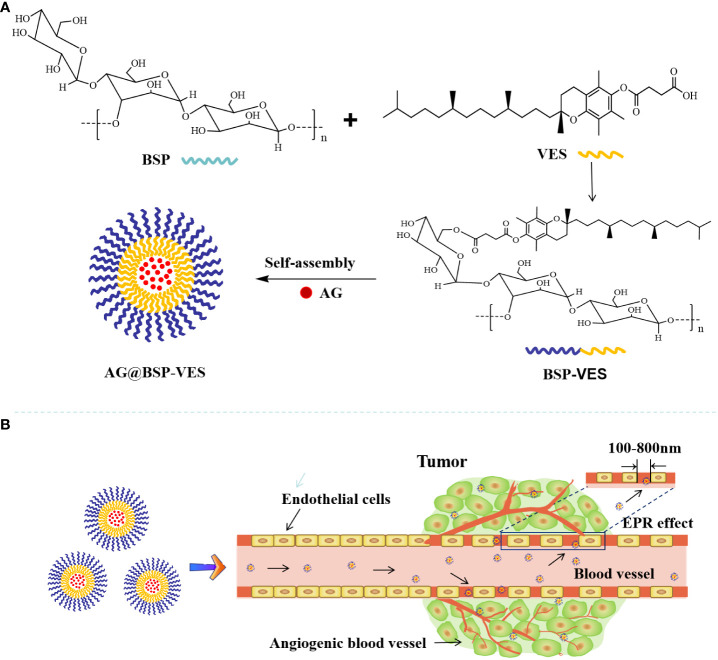
Schematic illustration of the delivery of andrographolide by BSP-VES polymer drug-loaded nano-micelles. **(A)** Synthesis of BSP-VES Polymers and self-assembly of BSP-VES Polymer micelles loaded with AG under Aqueous conditions; **(B)** Delivery of AG@BSP-VES micelles to tumors by passive targeting (EPR effect).

In conclusion, this study aims to issue the problems of poor water solubility, low bioavailability and short half-life *in vivo* of andrographolide (AG), and novel BSP combing with VES was used as the basic skeleton to construct polymer nano micellar carrier to deliver AG The drug delivery ability of BSP-VES *in vitro* and *in vivo* was evaluated. **Scheme 1** illustrates the process of synthesizing BSP-VES and then the delivery of AG into CT26 cells. The amphiphilic BSP-VES polymers are self-assembled into nano-micelles loaded with AG to enhance their selectivity and target the tumor site for precise therapy. This study develops a novel BSP-VES carrier to improve the therapeutic effect and survival rate of colon cancer patients, which could open up a new methodology for developing biomimetic nano-preparations from traditional Chinese medicines.

## 2 Materials and methods

### 2.1 Materials

*Bletilla striata* polysaccharide (BSP, purity > 95%, MW≈50000) was purchased from Shanghai Ronghe Pharmaceutical Technology Development Co., Ltd. (Shanghai, China). Vitamin E succinate (VES, purity 98%), andrographolide (AG, purity 98%), 1-yl-(3-dimethylaminopropyl) carbodiimide hydrochloride (EDC, purity 98%), 4-dimethylpyridine (DMAP, purity 99%), dimethyl sulfoxide (DMSO, purity 99%) and thiazolyl blue tetrazolium bromide (MTT, purity 98%) are all purchased from Shanghai Aladdin biochemical Technology Co., Ltd. (Shanghai, China). Fetal bovine serum (FBS) was purchased from Zhejiang Tianhang Biotechnology Co.,Ltd. (Hangzhou, China). RPMI. 1640 medium and PBS were purchased from Zhejiang Senrui Biotechnology Co., Ltd. (Hangzhou, China). 0.25% trypsin and penicillin-streptomycin solution were purchased from Gibco Company in the United States. Anhydrous ethanol with 99% purity was purchased from Chengdu Jinshan Chemical Reagent Co., Ltd. (Sichuan, China). Chromatographic methanol was purchased from Anhui Tiandi High Purity Solvent Co., Ltd. (Anhui, China); water is ultra-pure water; other reagents are analytical purity.

### 2.2 Cell lines and animals

The NCM460 cell (normal human colon epithelial cells), CT26-Luc (The luciferase-expressing mouse colon cancer cells) and CT26 (mouse colon cancer cells) were obtained from American Type Culture Collection (ATCC, Manassas, USA). These cell lines were cultured in RPMI- 1640, 10% FBS and 1% penicillin-streptomycin. BALB/c mice, SPF class, weighing 20 to 25 g, female, were purchased from Beijing Huafukang Biotechnology Co., Ltd. (certificate number: SCXK (Beijing) 2019–0008). Feeding conditions: the temperature is 22–25°C and the humidity is 40–70%. During the experiment, the animals were free to eat and drink, and the circadian rhythm was normal. The animal experiment was approved by the Animal Experimental Ethics Committee of Guizhou University of Traditional Chinese Medicine (Grant No.20220061).

### 2.3 Synthesis and characterization of BSP-VES polymer

Firstly, BSP-VES polymer was synthesized by esterification reaction in the presence of DMAP and EDC. Briefly, 202 mg of VES, 46.5 mg of DMAP, and 86.8 mg of EDC were dissolved in 15 mL of DMSO according to the ratio (N (VES):N(DMAP): N (EDC)= 1:1:1.2), then activated at 38°Cfor 1.5 h by stirring. Afterwards, 101 mg of BSP were dissolved in 25 mL of DMSO, and added into the mixture, which continued to react for 24 h. After reaction, 10 times the amount of ice anhydrous ethanol was added to the reaction solution, centrifuged and the acquired VES-BSP polymer was dried at 40°C. Then, the prepared VES-BSP polymer was dissolved in 8 mL of DMSO and transferred into a dialysis bag (Molecular weight cut-off, MWCO 3500) against deionized water for 48 h. The final BSP-VES polymer was obtained by freeze-drying.

BSP, VES, and BSP-VES structure were analyzed by 1H NMR on a Bruker Avance 500 (Billerica, MA, USA) with deuterated water (D2O) or DMSO. The BSP-VES polymer was diluted to 1 mg/mL with distilled water, and their particle size, PDI and zeta potential were measured using a DelsaMaxPRO Beckman nanoparticle size analyzer (Beckman, USA). The morphology of BSP-VES polymer was observed by transmission electron microscopy (TEM; JEOL JEM- 1230, Japan).

### 2.4 Determination of critical micelle concentration

The critical micelle concentration (CMC) of BSP-VES polymer was determined by fluorescence spectrometry, as described in the literature (29). Briefly, a pyrene solution with a concentration of 1.2 × 10–5 mmol/mL was prepared with methanol and placed in a brown volumetric flask, and the methanol was quickly evaporated by nitrogen. Various concentrations of BSP-VES solution (2.5, 10, 25, 100, 200, 400 µg/mL) were added to a pyrene methanol solution. Then, the resulting mixture was balanced for 24 h at 37°C. The emission spectra of a series of pyrene-containing BSP-VES solutions at 300–500 nm were scanned by fluorescence spectrophotometry. The first peak (I=374 nm) and the third peak (I=385 nm) of the fluorescence emission spectrum were recorded, and the ratio of the two peaks was calculated. Taking the logarithm of the BSP-VES concentration Lg C as the abscissa and I374/I385 as the ordinate, the experimental data were analyzed and fitted, and the BSP-VES concentration corresponding to the intersection of the curves was the CMC value.

### 2.5 Preparation and characterization ofAG@BSP-VES micelles

AG@BSP-VES was prepared by the dialysis method. Briefly, 29 mg of BSP-VES micelles were dissolved in ultrapure water. Then, 2.9 mg of AG was dissolved in absolute ethanol and added into the BSP-VES micelle solution. After stirring for 8 h, the micelle solution was transferred into a dialysis bag (3500 MWCO) against deionized water for 8 h. Finally, AG@BSP-VES was obtained through 0.45 microporous filter membrane.

Furthermore, High-performance Liquid Chromatography was used to evaluate the AG loading content and encapsulation efficiency in BSP-VES (HPLC, Agilent, China). Chromatographic conditions: C18 column (250 mm x 4.6 mm, 5 μm); The mobile phase was methanol-water solution (60:40) at a flow rate of 0.8 mL/min. The column temperature was 30°C. Detection wavelength was 226 nm and sample volume was 10 µL.

To draw the standard curve of AG, AG reference substance 10.1 mg was precisely weighed and diluted with methanol to the reference substance reserve solution of 0.202 mg/mL. Furthermore, 0.1, 0.5, 1, 2, 4, 6, 8 and 10 mL of the above solution were taken and supplemented with methanol to 10 mL, and then determined by HPLC. AG was extracted by organic solvent, and the content of AG was quantified by high-performance liquid chromatography. Encapsulation efficiency (EE) and drug loading (DL) were calculated by Equations 1 and 2, respectively.


(1) $$\text{EE}\left(\%\right)={\text{ C}}_{1}\;\times \;\text{V}/{\text{W}}_{1}\;\times \;100\;\%$$

(2) $$\text{DL}\left(\%\right){\text{= C}}_{1}{\;\times \text{V / (C}}_{1}{\;\times \text{V+W}}_{2}\text{) }\times 100 \%$$

In the formula, W_1_ and W_2_ represent the added mass of AG (mg) and the total mass of AG@BSP-VES (mg), respectively. C_1_ represents the drug concentration in methanol (μg/mL). V represents the added methanol volume (mL) when disrupt the nanomicelles.

### 2.6 The stability of AG@BSP-VES polymer

The stability of AG@BSP-VES polymer was further investigated by monitoring the changes in particle size, PDI and Zeta potential for 7 consecutive days using a Beckman nanoparticle size analyzer. During the experiment, the samples were stored at room temperature.

### 2.7 Hemolysis test

The hemocompatibility of the AG@BSP-VES micelle was evaluated by the erythrocyte hemolysis assay described in the literature (30, 31). Briefly, 2% (v/v) erythrocyte suspension obtained from the blood of rat was prepared by adding a certain amount of physiological saline. The AG@BSP-VES polymer was diluted with physiological saline to obtain solutions with concentrations of 0.172, 0.344, 0.685, 1.375, 2.750, 3.300, 4.240 and 5.500 mg/mL, respectively. Add 0.5 mL of AG@BSP-VES solution at various concentrations to a 2 mL EP tube containing 0.5 mL of erythrocyte suspension. The sample was shaken 30 min at 37°C, and centrifuged at 100 rpm for 15 min. Then, the supernatants of the samples in each group were collected. After that, the absorbance of supernatant at 540 nm was measured by the microplate reader. Physiological saline was used as a negative control group, and distilled water was used as a positive control group. The percentage of hemolysis was calculated through the formula below.


$$\text{Hemolysis }\left(\%\right){\text{ = (A}}_{\text{sample}}{\text{ - A}}_{\text{negative}}{\text{)/(A}}_{\text{positive}}{\text{ - A}}_{\text{negative}}\text{)}\times 100\%$$

### 2.8 *In vitro* drug release behavior study

The sample was transferred into a dialysis bag (MWCO 3.5 kDa), and then submerged into phosphate buffer PBS medium (pH=7.4) under the stirring (100 rpm) at 37°C. At the pre-designed time point (0, 0.5, 2, 4, 8, 12 and 24 h), 1 mL of release medium was taken out, and the fresh release medium of the same volume was supplemented. The released AG in the medium was determined by HPLC, and the cumulative release of the AG was calculated according to the standard curve.

### 2.9 Cellular uptake study

NCM460 or CT26 cells in the logarithmic growth phase, were digested with 0.25% trypsin into a single cell suspension. The suspension was then diluted to a concentration of 2×105 cells/mL using RPMI- 1640. Then, a 1-mL cell suspension was seeded into a 24-well cell plate and placed in an incubator at 37°C with 5% CO2 for 24 h. After that, the Coumarin 6 (C6)-labeled AG@BSP-VES (AG, 2μg/mL) was diluted to a concentration of 1 mg/mL and added to a 24-well plate in a time gradient 1, 2, 4, 12 and 24 h. For investigating the targeted cellular uptake of AG@BSP-VES, CT26 and NCM460 cells were incubated with AG@BSP-VES (AG, 2μg/mL) for 2 h. At the end of the incubation period, the supernatant was removed and washed twice with PBS to eliminate any residual serum and free drug interfering substances (32). Finally, the cellular fluorescence was examined using a fluorescence microscope (Leica DMIL LED; Leica, Solms, Germany). Furthermore, the cellular fluorescence intensity was determined through the Image J Software.

### 2.10 *In vitro* biosafety evaluation

The *in vitro* biosafety of BSP-VES was evaluated using the thiazolyl blue tetrazolium bromide (MTT) assay. NCM460 or CT26 cells were seeded in 96-well plates and treated with BSP-VES at concentrations of 0.125, 0.25, 0.5, 0.7, 0.8, and 1 mg/mL for 24 or 48 h. Untreated blank cells were used as the control. Then, 20 µL of MTT (5 mg/mL) was added to each well and cultured for 4 h in the incubator. Afterward, the supernatants were removed, and 100 μL of dimethyl sulfoxide (DMSO) was added to dissolve the formazan crystals. The absorbance of cells at 570 nm was measured using a Microplate Reader (Thermo, Multiskan Sky, USA), and the cell proliferation rate was calculated using the provided formula.


$${\text{Cell viability = (OD}}_{\text{sample}}{\text{ - OD}}_{\text{blank}}{\text{)/(OD}}_{\text{control}}{\text{ - OD}}_{\text{blank}}\text{) }\times 100\%$$

### 2.11 *In vitro* antitumor activity studies

The *in vitro* antitumor activity of AG@BSP-VES was evaluated by MTT assay, the fluorescent live/dead (33) and the scratch wound assay (34). For the MTT assay, CT26 cells were initially inoculated into a 96-well plate at a density of 5×104/mL. Following a 24-hour incubation period, AG and AG@BSP-VES were added to the plate at various concentration gradients from 0.09 to 22 µg/mL, while untreated cells were used as a control. The absorbance was then measured at 570 nm on a Microplate Reader. For the fluorescent live/dead assay, NCM460 or CT26 cells in logarithmic growth phase were inoculated on 96-well plate and cultured for 24 h. Then, AG and AG@BSP-VES solutions were added at a same drug concentration of 15 µg/mL. Besides, the untreated blank cells were used as control. Post-culture, cells were washed twice with 100 µL PBS, and LIVE/DEAD Near-IR Fixable dye was added, cultivated continuously for 20 minutes, and then imaged under a fluorescence microscope. Calcein AM and ethidium homodimer- 1 were used to stain living and dead cells, respectively. The number of live and dead cells was measured by Image J software. For the scratch wound assay, CT26 cells were divided into 24-well plates at a cell density of 10× 105 cells/mL, and cultured for 24 h to form a monolayer of cells. When the density reached 90%, a 10 µL pipette tip was adopted to draw a line, and washed with PBS for two times to remove cell debris. Appropriate concentrations of AG and AG@BSP-VES were added to a 24-well plate. The migration and invasion of CT26 cells were observed under a microscope at four different time points (0 h, 6 h, 12 h, 24 h). The scratch area was determined using Image J software, and the average value was measured three times simultaneously. The cell migration rate was calculated using the formula (%) = (0 h scratch width − 24 h scratch width)/0 h scratch width × 100%.

### 2.12 *In vivo* imaging studies

All animal studies were conducted under Institutional Animal Care and Use Committee-approved protocols of Guizhou University of Traditional Chinese Medicine. In order to investigate the *in vivo* distribution, AG@BSP-VES micelles (AG, 10 mg/kg) were further labeled with DIR (a near infrared fluorescent fuel). The subcutaneous (CT26 cells) and *in situ* (CT26-Luc cells with the expression of insect luciferase) tumor models were established based on previous studies (35). Briefly, BABL/C mouse were anesthetized and its abdominal cavity was cut longitudinally to expose colon tissues. CT26-luc cells (5×10^6^ cells in 20 μL PBS) were injected to the colorectal membrane. The wound of mouse was closed by biodegradable stitches. Four days later, the growth of the tumors was monitored by the IVIS Lumina II *in vivo* imaging system (Caliper Life Science, USA) after an injection of luciferin. To further confirm the establishment of orthotopic colon tumors, mice were killed, and colon tissues bearing tumors were stripped and immediately fixed in 4% (vol/vol) formaldehyde, which were evaluated by hematoxylin and eosin (H&E) and Ki67 staining. AG@BSP-VES were injected into CT26 tumor-bearing mice by intravenous injection. The mice were observed by the IVIS Lumina II *in vivo* imaging system at the predetermined time (1 h, 2 h, 4 h, 8 h, 12 h, and 24 h) after the injection. After that, the mice were euthanized by vein air injection. Various tissues, including tumors, were collected, weighted and observed by the *in vivo* imaging system. The accumulation of AG@BSP-VES micelles in various tissues was calculated as %ID/g (the percentage of the injected dose per gram of tissue).

### 2.13 Statistical analysis

Student’s t-test was used to determine the statistical significance of differences between groups. For all analyses, statistical significance was set at P < 0.05 for each paired experiment.

## 3 Results

### 3.1 Synthesis and characterization of BSP-VES polymer

**Figure 1:**
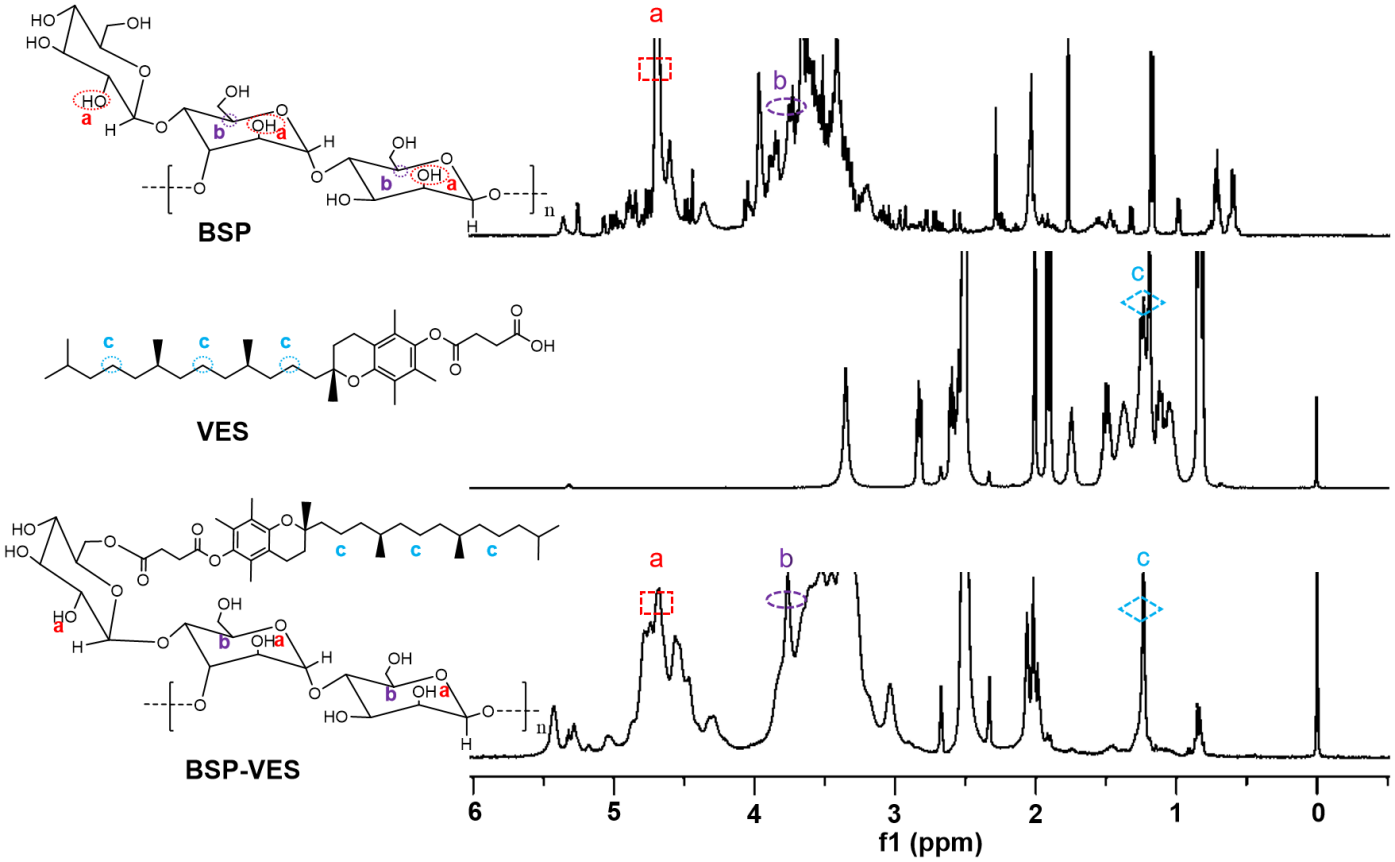
^1^H NMR spectra of BSP, VES, BSP-VES from top to bottom, respectively.

Amphiphilic BSP-VES polymer was synthesized by esterification and confirmed by 1H NMR (**Figure 1**). The 1H NMR spectra of BSP, VES and BSP-VES were presented in this order from top to bottom. As shown in **Figure 1**, BSP has two characteristic peaks at approximately 3.74–3.8 ppm and 4.66–4.80 ppm, which corresponds to the methine and hydroxyl proton peak of the simple sugar molecule in BSP. VES has a single peak at 1.21- 1.26 ppm, which can be attributed to the proton peak of methylene on the long chain of VES. All different characteristic peaks from a-c in BSP and VES appeared in the 1H NMR spectrum of BSP-VES, which indicates that BSP-VES polymer has been successfully synthesized.

**Figure 2:**
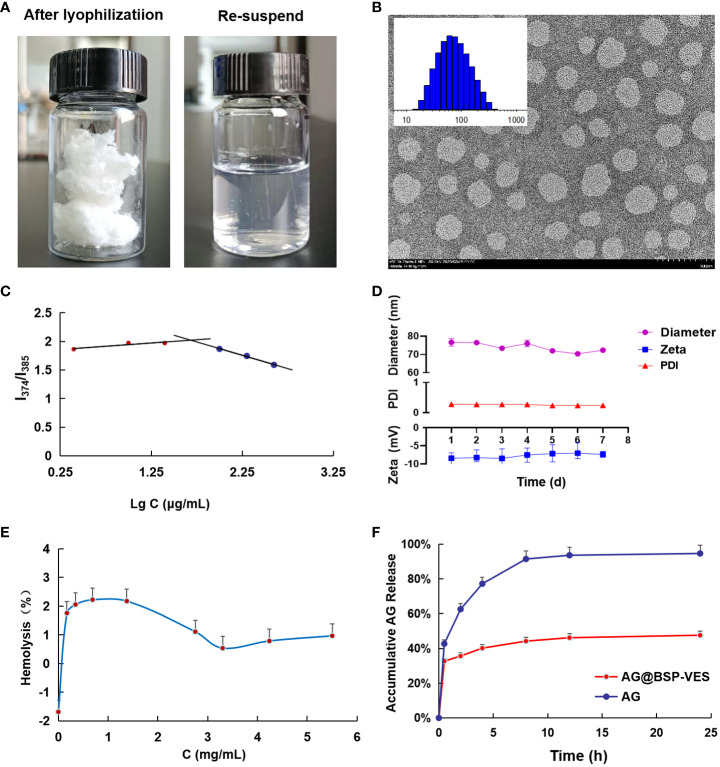
Characterization of BSP-VES and AG@BSP-VES. **(A)** The powder of AG@BSP-VES micelles (after lyophilization) and the solution containing AG@BSP-VES micelles (the re-suspended). **(B)** Particle size distribution and TEM image of BSP-VES micelles. The samples were stained by phosphotungstic acid (2% W/V) (× 100,000, bar =100 nm). **(C)** Variation of intensity ratio (I374/I385) vs concentration of BSP-VES. **(D)** Stability assay of AG@BSP-VES micelles in PBS during 7-day storage at room temperature (n=5). **(E)** The hemolysis rate of AG@BSP-VES (n=3). **(F)** The release of AG from AG@BSP-VES micelles for 24 h (n=3).

The average diameter and zeta potential of the BSP-VES polymer micelle were 65.34 ± 1.03 nm and -9.12 ± 0.61 mV, respectively. As shown in **Figure 2B**, the average diameter of BSP-VES was nearly consistent with the TEM results. In addition, BSP-VES polymer showed excellent dispersity and ability to self-assemble into nano micelles in aqueous environments with the critical micelle concentration (CMC) of 48.3 μg/mL (**Figure 2C**).

### 3.2 Preparation and characterization of AG@BSP-VES micelles

AG@BSP-VES micelles were successfully prepared by the dialysis method, presenting a circular specific core-shell structure. The average diameter of AG@BSP-VES micelles increased from 65.34 ± 1.03 nm to 84.68 ± 2.08 nm because of the loading of AG, as determined using dynamic light scattering (**Table 1**). In addition, AG@BSP-VES micelles showed good stability. As shown in **Figure 2D**, after AG@BSP-VES micelles were placed at room temperature for 7 days, no significant changes were observed in particle diameter, Zeta Potential and PD Index value. Hemolysis is an essential preliminary assessment of the toxic effects of biological materials. As shown in **Figure 2E**, the hemolysis rate is 1%-3% (less than 5%), which has good blood compatibility and was coincident with the criteria of the AG@BSP-VES micelles could easily disperse in the aqueous medium to form the micelles again, which indicated that AG@BSP-VES micelles have higher physical-chemical stability before the injection for the administration (**Figure 2A**).

**Table 1:** Particle diameter, PDI and Zeta Potential of BSP-VES and AG@BSP-VES (n = 5).

Sample	Diameter(nm)	PDI (-)	Zeta Potential (mV)
BSP-VES	65.34 ± 1.03	0.242 ± 0.005	-9. 12 ± 0.61
AG@BSP-VES	84.68 ± 2.08	0.241 ± 0.006	-12.49 ± 2.28

According to the above chromatographic conditions, linear regression was performed on the peak area (Y) and concentration (X), and the linear regression equation was obtained as Y=20. 133X+14. 153 (R2 = 0. 9999). The results showed that AG had a good linear relationship in the range of 2.02–202 μg/mL. Based on the linear regression equation, the entrapment efficiency of AG@BSP-VES was found to be 91. 15 ± 0.05%, with a drug loading of 6.84 ± 0.01%. These results suggest that the synthesized micelles exhibit a high drug-loading capacity. **Figure 2F** shows the characteristics of AG release from AG@BSP-VES micelles. As shown in **Figure 2F**, almost all of the free AG was released within 8 h. However, AG release from the micelles was significantly slow, with only about 47.6% AG accumulative release after 24 h, defined as the percentage of released AG to total entrapped AG. The result showed that AG@BSP-VES micelles had a good sustained release effect. (PS. The initial rapid release of AG@BSP-VES may be attributed to the quick release of drugs that are embedded or adsorbed on or near the surface of the particles within a short periodof time).

### 3.3 *In vitro* cellular uptake and biosafety studies

**Figure 3:**
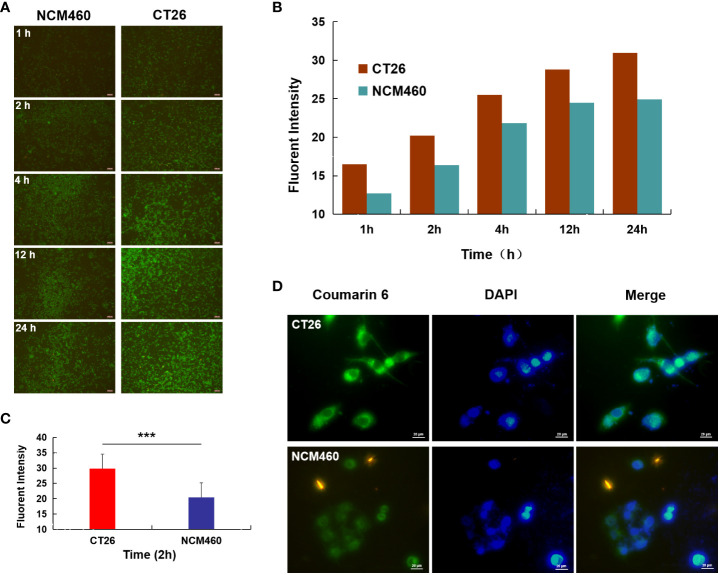
Cellular uptake studies. **(A)** Fluorescence image of CT26 and NCM460 cells incubated with AG@BSP-VES for 1, 2, 4, 12 and 24 h, respectively; **(B)** The quantitative analysis based on the imaging in **(A)** by a software “Image J”. **(C)** Cell uptake of AG@BSP-VES (Coumarin 6 labeled) in CT26 and NCM460 cells with 2h incubation time. **(D)** The quantitative analysis based on the imaging in **(C)** by a software “Image J”. (mean ± SD, n=3, ***P < 0.001).

As shown in **Figure 3A**, significantly more AG@BSP-VES micelles were internalized into CT26 cells compared with NCM460 cells during whole incubation time (i.e. 24 h), which was confirmed quantitatively by Image J software (**Figure 3B**). As shown by further targeted cellular uptake result, the average fluorescent intensity of AG@BSP-VES micelles in CT26 cells after 2 h incubation, was significantly higher than that in NCM460 cells (**Figures 3C, D**).

**Figure 4:**
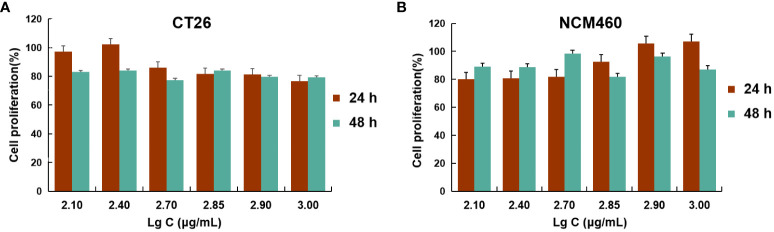
Cell viability studies. CT26 **(A)** and NCM460 **(B)** cells were incubated with different concentrations of BSP-VES for 24 h and 48 h. Cell viability was calculated by the percentage of living cells (n=4).

These data demonstrated that AG@BSP-VES micelles had potential colon cancer cell specificity. The *in vitro* bio-safety of BSP-VES micelles against CT26 and NCM460 cells was investigated by MTT assay. As shown in **Figures 4A, B**, blank BSP-VES micelles showed good biosafety during the period of incubation. The cell viabilities of CT26 and NCM460ncells were almost all over 80% after 24 or 48 h of incubation.

### 3.4 *In vitro* cytotoxicity study

**Figure 5:**
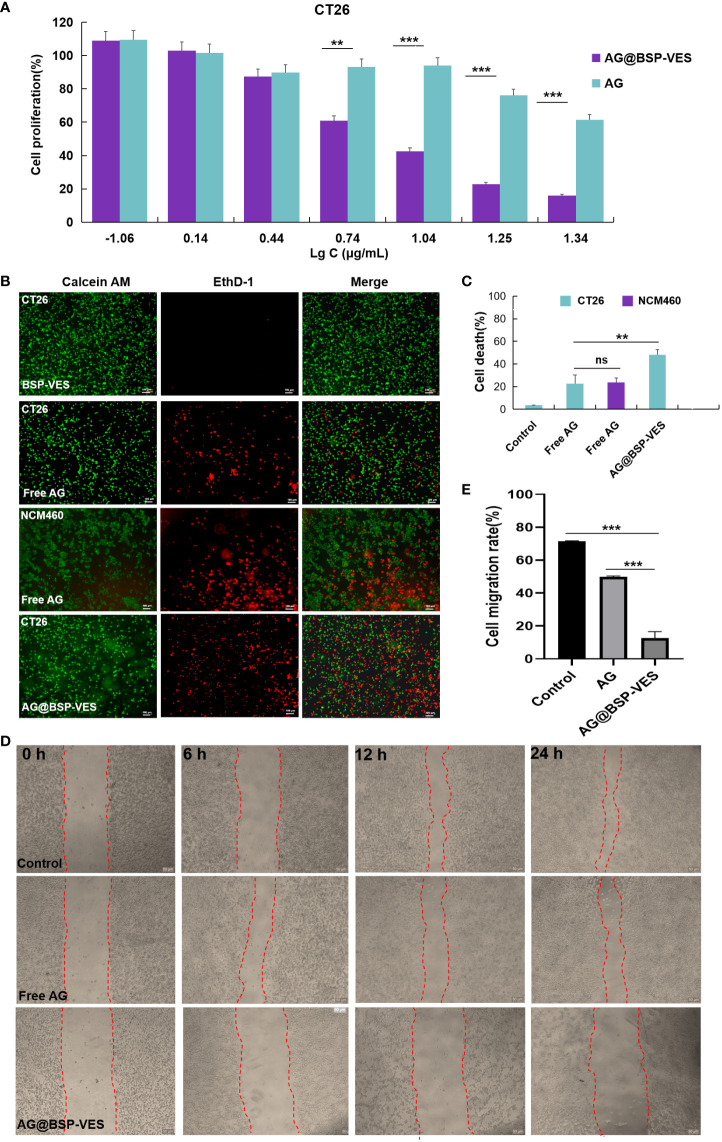
*In vitro* anti-tumor activity studies. **(A)** CT26 and NCM460 cells treated with AG and AG@BSP-VES. **(B)** CT26 and NCM460 cells treated with BSP-VES, AG and AG@BSP-VES. Cells were stained with calcein AM (living cells, green) and EthD- 1 (dead cells, red). **(C)** The quantitative analysis based on the imaging in **(B)** by a software “Image J “. **(D)** The scratch wound assay in CT26 cells with PBS, AG and AG@BSP-VES. **(E)** The quantitative analysis based on the imaging in **(D)** by a software “Image J“. (mean ± SD, n=3, **P <0.01, ***P < 0.001).

**Figure 5A** shows the cytotoxic effects of free AG and AG@BSP-VES in CT26 cells. The significantly higher cell-killing efficiency of AG@BSP-VES micelles than that of free AG alone was presented (*P*<0.001). Over 75% of CT26 cells were killed after 24 h incubation with AG@BSP-VES micelles (17.6 µg/mL). However, under the same AG concentration, only 23.9% of CT26 cells were killed. IC50 values were respectively 11.69 and 31.16 µg/mL for CT26 cells treated with AG@BSP-VES micelles and free AG. It was mainly attributed to the excellent internalization and tumor specificity of AG@BSP-VES micelles, resulting in increased AG concentration in CT26 cells. In addition, significantly more CT26 cells were killed (stained red by EthD- 1) due to the enhanced chemotherapeutical effect of AG from AG@BSP-VES micelles (the cell death percentage of 52.78%), compared with free AG group (the cell death percentage of 24.46%), which was attributed to more AG@BSP-VES micelles were internalized into CT26 cells than the free AG group (**Figures 5B, C**). Due to lack of selectivity, free AG group had induced similar cell death in both CT26 (24.46%) and NCM460 (26.25%) cells. The wound healing assay was used to investigate anti-invasion and anti-migration effects of AG@BSP-VES micelles. As shown in **Figures 5D, E**, the scratch area of AG@BSP-VES micelles group (71.40 ± 0.43%) measured by ImageJ software was significantly larger than that of free AG group (49.8 ± 0.56%). In addition, the percentage of scratch area was significantly decreased by 12.6 ± 3.95% in control group. These results showed that AG@BSP-VES micelles exhibited an excellent inhibitory effect on CT26 cells, compared with free AG, which was a direct result of BSP-VES micelles effectively increased cellular uptake of AG into CT26 cells and enhanced sensibility of CT26 cells to AG.

### 3.5 *In vivo* imaging

**Figure 6:**
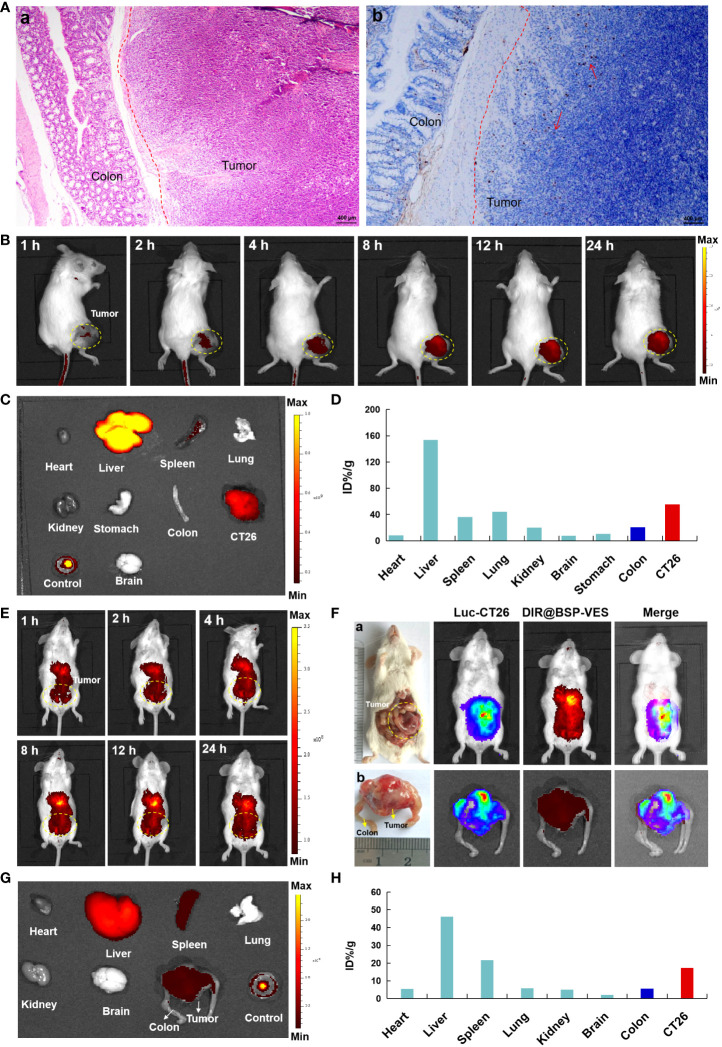
The *in vivo* imaging studies of DIR labeled AG@BSP-VES micelles. **(A)** The establishment of orthotopic CT26-Luc tumor mouse model. The tumor-colon tissue were stained with H&E (a) and Ki67 (b) stain. **(B)** The *in vivo* imaging of the mice, bearing subcutaneous tumors, at different time after iv injection of the micelles. **(C)** The fluorescent imaging of various tissues in subcutaneous model at 24 h after the iv injection of the micelles. **(D)** The accumulation of the micelles in various tissues of **(B)** was calculated as %ID/g. **(E)** The *in vivo* imaging of the micelles in *in situ* CT26-Luc tumors at different time after iv injection. **(F)** The *in vivo* imaging of the micelles in *in situ* CT26-Luc tumors at 24 h after iv injection (5 min after IP injection of D-luciferin). (a) The anatomy and *in vivo* imaging of CT26-Luc tumor-bearing mice. (b) The photograph and *in vivo* imaging of CT26-Luc tumor and normal colon tissues. **(G)** The fluorescent imaging of various tissues at 24 h after the iv injection of the micelles. **(H)** The accumulation of the micelles in various tissues of **(F)** was calculated as %ID/g (the percentage of the injected dose per gram of tissue).

In order to further investigate the targeted accumulation of AG@BSP-VES micelles in CT26 tumors, we simultaneously established the subcutaneous and *in situ* tumor models. As shown in **Figure 6A**, the dividing line tumor and colon tissue is clearly visible, and tumor cells are marked by the tendency to spread, especially into normal colon tissue (**Figure 6B**, cells in proliferation was stained (brown) by Ki67 stain). With the increase of the experiment time, the accumulation of AG@BSP-VES micelles in subcutaneous CT26 tumors showed an obvious increase tendency (**Figure 6B**). The fluorescent intensity of various tissues after 24 h injection was observed (**Figure 6C**), and quantitated. There was the highest accumulation of AG@BSP-VES micelles at 24 h after injection in subcutaneous CT26 tumors (55.37%, ID/g) and normal colon tissues (20.44%, ID/g) tumors (**Figure 6D**). Surprisingly, the targeted accumulation effect of AG@BSP-VES micelles was also observed in *in situ* CT26-Luc tumors (**Figures 6E–H**). There was the highest accumulation of AG@BSP-VES micelles at 24 h after injection in CT26-Luc tumors (17.31%, ID/g) and normal colon tissues (5.66%, ID/g). Interestedly, the bioluminescence signal of CT26-Luc was almost merged with the fluorescent signal of AG@BSP-VES micelles (**Figure 6F**). These results indicated that AG@BSP-VES could effectively and specifically internalize into colon tumors.

## 4 Discussion

Recently, the incidence of colon cancer has increased due to the growth of the economy and the rise of dietary fat content, of fat composition in dietary fat content, with the age of diagnosis decreasing sharply over time (36). Surgery combined with radiation and chemotherapy is the current approach for treating colon cancer; however, it can result in recurrence and metastasis after surgery. Mostly, the biological distribution of chemotherapeutic drugs to tumors is poor, and the high-dose chemotherapy is toxic to normal tissue. Therefore, the development of targeted carriers to deliver drugs with antineoplastic activity can improve the therapeutic index of chemotherapy, while reducing the toxicity of chemotherapy to other tissues.

BSP is a hydrophilic polymer, that can be hydrophobically modified by VES to form an amphiphilic polymer. The synthesis of BSP-VES polymer was confirmed by 1H NMR (**Figure 1**). These amphiphilic polymers have the ability to self-assemble in water, forming micelles with core- shell structure and can be loaded with AG. The critical micelle concentration, particle size, PDI, and TEM morphology were analyzed, revealing that the synthesized BSP-VES polymer micelles (with a particle size smaller than 100 nm) exhibit favorable properties (**Figures 2A, B**, **Table 1**). When compared to synthetic polymer micelles, natural polymer micelles are considered safer due to their bio-compatibility and ease of degradation. In this study, we evaluated the *in vitro* biosafety of BSP-VES and found that BSP-VES exhibited good biosafety (**Figure 4**). Polymer micelles are known to be thermodynamically stable assemblies, and we investigated the stability of AG@BSP-VES and the results showed that the particle size, potential, and PDI of the micelles remained relatively unchanged for up to 7 days (**Figure 2E**). Meanwhile, AG@BSP-VES showed a good sustained release effect and good blood compatibility, and it was coincident with the criteria of the medical devices (**Figures 2E, F**). We also examined the BSP-VES, which forms a micelle with a hydrophilic shell and hydrophobic core when introduced to water. It has been shown that the hydrophilic shell of the micelle can effectively prevent the drug loading system from being non-specifically recognized and swallowed by the reticuloendothelial system (RES), thereby significantly prolonging the drug’s half-life (37). Furthermore, micelles ranging in particle size from 10 to 100 nm have the ability to evade phagocytosis by the reticuloendothelial system (RES) and clearance by the mononuclear phagocytic system (MPS), resulting in a longer systemic circulation time. This, in turn, allows for passive targeting of tumors by enhancing the permeability and retention effect (EPR effect), ultimately improving the therapeutic outcome (38). In addition, the particle size of AG@BSP-VES (84.68 ± 2.08 nm, less than 100 nm) became bigger compared with BSP-VES micelles (65.34 ± 1.03 nm) (**Figure 2C** and **Table 1**), which might be due to the larger volume of micelles after the hydrophobic micelle core was loaded with the drug (39). The result suggested that the AG@BSP-VES has the potential to effectively target tumors. Tumor lesions are characterized by fast-growing cells that destroy the inner wall of tissue blood vessels, resulting in increased permeability compared to normal tissue. Therefore, self-assembled micelles can easily internalize into tumor cells, or penetrate and remain in the tumor tissue (40). In addition, *Bletilla striata* polysaccharide contain abundant mannose units, which exhibit a strong affinity to mannose receptors on macrophages. Therefore, *Bletilla striata* polysaccharide based nanocarrier system could be captured by the reticuloendothelial system through endocytosis or fusion mediated by mannose receptors after the system enters the systemic circulation, and then concentrates in the liver, kidneys, spleen and other organs to achieve active targeting (41). Zhang et al. (42) prepared the ALN-BSP by covalently linking the bonds of both substances (i.e. *Bletilla striata* polysaccharide-alendronate and alendronate sodium). ALN-BSP nanoparticles are rapidly taken up by cells and delivered to tumor tissues driven by the EPR effect through endocytosis mediated by mannose receptor.

It explained why AG@BSP-VES could more internalized into CT26 cells but NCM460 cells during whole incubation time (i.e. 24 h) (**Figure 3**). Meanwhile, the targeted accumulation effect of AG@BSP-VES micelles could also be observed in subcutaneous and *in situ* tumors (**Figure 6**). For these reasons, AG@BSP-VES micelles exhibited more excellent inhibitory effect on CT26 cells compared with free AG. The IC50 values of AG@BSP-VES micelles (31. 16 µg/mL) were more than 3 times greater than free AG (11.69 µg/mL) (**Figure 5A**). Encouragingly, the significantly higher cell-killing effects of AG@BSP-VES micelles on CT26 cells were also observed in the fluorescent live/dead and scratch wound experiments (**Figures 5B–E**), which could be attributed to various factors, including the EPR effect, the affinity of carriers. In addition, VES has the role of immune regulation and, to some extent, plays a synergistic role with anti-tumor drugs in the treatment of colon cancer (43–46). Therefore, the tumor-targeting and synergistic anti-tumor mechanism of AG@BSP-VES micelles would be the next research points.

## 5 Conclusions

This work design and synthesize an amphiphilic BSP-VES polymeric micelle to enhance the bioavailability and tumor targeting of AG. The findings of this study suggest that the synthetic carrier has a low hemolysis rate and exhibits excellent biological safety with no significant effect on various cell lines. Furthermore, the AG delivered through the BSP-VES vector has shown an enhanced suppression effect on colon cancer cells (CT26) compared to free AG, indicating that BSP-VES improves the bioavailability of AG. Compared with free AG, the enhanced cytotoxicity of AG@BSP-VES micelles against tumor cells can be attributed to the increased cellular uptake of AG mediated by BSP-VES micelles and the enhanced chemotherapeutic effect of AG released from the micelles. Our data also demonstrated that the specific delivery of AG@BSP-VES micelles into subcutaneous and *in situ* tumors was achieved after iv administration during the whole experiment process. Therefore, BSP-VES micelles have the potential to be a promising drug carrier for efficient delivery of hydrophobic drugs and targeted treatment of colon cancer.

## Data availability statement

The datasets presented in this study can be found in online repositories. The names of the repository/repositories and accession number(s) can be found in the article/supplementary material.

## Ethics statement

All experimental procedures presented in this manuscript were approved and carried out in accordance with the China Veterinarian Animal Care Office Animal Experimental Ethics Committee of Guizhou University of Traditional Chinese Medicine (Grant No.20220061).

## Author contributions

ZY: Conceptualization, Writing – review & editing, Formal analysis, Software, Writing – original draft. YUZ: Formal analysis, Software, Writing – original draft, Methodology, Project administration. TC: Methodology, Conceptualization, Funding acquisition, Writing – review & editing. TF: Funding acquisition, Formal analysis, Project administration, Writing – original draft. YIZ: Funding acquisition, Conceptualization, Resources, Writing – review & editing. JZ: Conceptualization, Writing – review & editing, Methodology. NZ: Writing – review & editing, Formal analysis, Validation. JY: Validation, Writing – review & editing. GL: Writing – review & editing, Software. ZW: Writing – review & editing, Conceptualization, Funding acquisition, Project administration, Resources, Validation.
